# Bis[3-(pyrazin-2-yl)-5-(pyridin-2-yl-κ*N*)-1,2,4-triazol-1-ido-κ*N* ^1^]copper(II)

**DOI:** 10.1107/S1600536812016777

**Published:** 2012-04-25

**Authors:** Lin-Lin Cong, Min-Yan Kang, Yu-Fei Ji, Zhi-Liang Liu

**Affiliations:** a College of Chemistry and Chemical Engineering, Inner Mongolia University, Hohhot, People’s Republic of China

## Abstract

In the mononuclear title complex, [Cu(C_11_H_7_N_6_)_2_], the Cu^II^ atom lies on a crystallographic inversion centre and is coordinated by four N atoms from two bidentate chelate monoanionic 3-(pyrazin-2-yl)-5-(pyridin-2-yl-1,2,4-triazol-1-ido ligands, two from the triazolide rings [Cu—N = 1.969 (2) Å] and two from the pyridine rings [Cu—N = 2.027 (2) Å], giving a slightly distorted square-planar geometry.

## Related literature

For details of the synthesis and properties of related copper compounds showing a similar coordination environment, see: Meng *et al.* (2009 ▶); Cheng *et al.* (2007 ▶); Zhang *et al.* (2005 ▶). For the structure of an Ru^II^ complex with the same ligand, see: Browne *et al.* (2002 ▶).
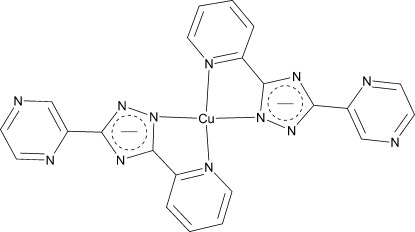


## Experimental

### 

#### Crystal data

[Cu(C_11_H_7_N_6_)_2_]
*M* *_r_* = 509.99
Monoclinic, 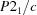

*a* = 11.9735 (4) Å
*b* = 10.7539 (3) Å
*c* = 8.0162 (3) Å
β = 106.500 (4)°
*V* = 989.67 (6) Å^3^
*Z* = 2
Mo *K*α radiation
μ = 1.15 mm^−1^
*T* = 293 K
0.20 × 0.20 × 0.20 mm

#### Data collection

Bruker SMART APEX diffractometer
Absorption correction: multi-scan (*SADABS*; Sheldrick, 1996 ▶) *T*_min_ = 0.795, *T*_max_ = 0.795
3248 measured reflections
1739 independent reflections
1451 reflections with *I* > 2σ(*I*)
*R*_int_ = 0.021

#### Refinement

*R*[*F* ^2^ > 2σ(*F* ^2^)] = 0.033
*wR*(*F* ^2^) = 0.083
*S* = 1.06
1739 reflections
160 parameters
H-atom parameters constrained
Δρ_max_ = 0.28 e Å^−3^
Δρ_min_ = −0.27 e Å^−3^

### 

Data collection: *SMART* (Bruker, 2001 ▶); cell refinement: *SAINT* (Bruker, 2001 ▶); data reduction: *SAINT*; program(s) used to solve structure: *SHELXS97* (Sheldrick, 2008 ▶); program(s) used to refine structure: *SHELXL97* (Sheldrick, 2008 ▶); molecular graphics: *DIAMOND* (Brandenburg & Putz, 2006 ▶); software used to prepare material for publication: *publCIF* (Westrip, 2010 ▶).

## Supplementary Material

Crystal structure: contains datablock(s) I, global. DOI: 10.1107/S1600536812016777/zs2193sup1.cif

Structure factors: contains datablock(s) I. DOI: 10.1107/S1600536812016777/zs2193Isup2.hkl

Additional supplementary materials:  crystallographic information; 3D view; checkCIF report

## Figures and Tables

**Fig. 1.**
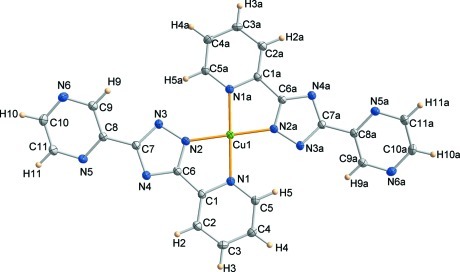
The molecular structure of the title complex, showing 30% probability displacement ellipsoids. For symmetry code (a): -x + 2, -y + 2, -z.

## supplementary crystallographic information

### Comment

2-(5-Pyridin-2-yl)-1,2,4-triazol-3-yl)pyrazine (Hptp) is a potentially multidentate ligand containing multiple *N* coordination sites. The synthesis and properties of copper complexes with similar ligands have been described (Meng *et al.*, 2009; Cheng *et al.*, 2007; Zhang *et al.*, 2005). However, only one crystal structure of a metal complex with the named ligand has been reported in the crystallographic literature, that with Ru (Browne *et al.*, 2002). Herein, we report the synthesis and crystal structure of the copper(II) complex with the ptp^-^ ligand, the title complex [Cu(C_11_H_7_N_6_)_2_].

As shown in Fig. 1, this complex is a discrete neutral monomer, in which the Cu^II^ atom resides on a crystallographic inversion centre. The Cu^II^ atom is in a slightly distorted [N_4_] square planar environment, with the coordination sphere defined by two pyridyl N-atom donors [Cu—N_pyridine_ = 2.027 (2) Å] and two triazolate N-atom donors [Cu—N_triazolide_ = 1.969 (2) Å] from two bidentate chelate ptp^-^ anion ligands. The dihedral angle between the coordinated pyridyl group and the triazolato ring is 1.33 (9)°. In the crystal packing there are only minor weak intermolecular C—H···N hydrogen-bonding interactions (Table 1).

### Experimental

CuCl_2 ` _2H_2_O (1 mmol, 0.1704 g), Hptp (1 mmol, 0.2242 g), aqueous ammonia (25%, 2.0 ml) and water (15 ml) were heated in a 23-ml Teflon-lined autoclave at 160 °C for 3 days, followed by slow cooling (5 °C h^-1^) to room temperature. The black block crystals were filtered off and washed with water (yield 42%, based on CuCl_2 ` _2H_2_O). IR (KBr, cm^-1^): 3424 (br), 3048 (w), 1620 (*s*), 1465 (*s*), 1401 (*m*), 1377 (*m*), 1120 (w), 1026 (w), 763 (*m*).

### Refinement

All H atoms were placed in geometrically idealized positions and constrained to ride on their parent atoms with C—H = 0.93 Å and with *U*_iso_(H) = 1.2*U*_eq_(C).

### Crystal data

**Table:**

[Cu(C_11_H_7_N_6_)_2_]	*F*(000) = 518
*M**_r_* = 509.99	*D*_x_ = 1.711 Mg m^−^^3^
Monoclinic, *P*2_1_/*c*	Mo *K*α radiation, λ = 0.71073 Å
Hall symbol: -P 2ybc	Cell parameters from 2919 reflections
*a* = 11.9735 (4) Å	θ = 2.6–25.0°
*b* = 10.7539 (3) Å	µ = 1.15 mm^−^^1^
*c* = 8.0162 (3) Å	*T* = 293 K
β = 106.500 (4)°	Block, black
*V* = 989.67 (6) Å^3^	0.20 × 0.20 × 0.20 mm
*Z* = 2	

### Data collection

**Table:**

Bruker SMART APEX I diffractometer	1739 independent reflections
Radiation source: fine-focus sealed tube	1451 reflections with *I* > 2σ(*I*)
Graphite monochromator	*R*_int_ = 0.021
Detector resolution: 8.192 pixels mm^-1^	θ_max_ = 25.0°, θ_min_ = 2.6°
ω–2θ scans	*h* = −14→13
Absorption correction: multi-scan (*SADABS*; Sheldrick, 1996)	*k* = −11→12
*T*_min_ = 0.795, *T*_max_ = 0.795	*l* = −9→6
3248 measured reflections	

### Refinement

**Table:**

Refinement on *F*^2^	Primary atom site location: structure-invariant direct methods
Least-squares matrix: full	Secondary atom site location: difference Fourier map
*R*[*F*^2^ > 2σ(*F*^2^)] = 0.033	Hydrogen site location: inferred from neighbouring sites
*wR*(*F*^2^) = 0.083	H-atom parameters constrained
*S* = 1.06	*w* = 1/[σ^2^(*F*_o_^2^) + (0.0356*P*)^2^ + 0.5521*P*] where *P* = (*F*_o_^2^ + 2*F*_c_^2^)/3
1739 reflections	(Δ/σ)_max_ < 0.001
160 parameters	Δρ_max_ = 0.28 e Å^−^^3^
0 restraints	Δρ_min_ = −0.27 e Å^−^^3^

### Special details

**Table:**

Geometry. Bond distances, angles etc. have been calculated using the rounded fractional coordinates. All su's are estimated from the variances of the (full) variance-covariance matrix. The cell esds are taken into account in the estimation of distances, angles and torsion angles
Refinement. Refinement of *F*^2^ against ALL reflections. The weighted *R*-factor *wR* and goodness of fit *S* are based on *F*^2^, conventional *R*-factors *R* are based on *F*, with *F* set to zero for negative *F*^2^. The threshold expression of *F*^2^ > σ(*F*^2^) is used only for calculating *R*-factors(gt) *etc*. and is not relevant to the choice of reflections for refinement. *R*-factors based on *F*^2^ are statistically about twice as large as those based on *F*, and *R*- factors based on ALL data will be even larger.

### Fractional atomic coordinates and isotropic or equivalent isotropic displacement parameters (Å^2^)

**Table:**

	*x*	*y*	*z*	*U*_iso_*/*U*_eq_	
Cu1	1.00000	1.00000	0.00000	0.0286 (2)	
N1	1.10513 (18)	0.85051 (18)	0.0123 (3)	0.0267 (7)	
N2	0.91236 (19)	0.87911 (18)	0.0980 (3)	0.0288 (7)	
N3	0.81241 (19)	0.87336 (18)	0.1478 (3)	0.0298 (7)	
N4	0.88670 (18)	0.67872 (18)	0.1531 (3)	0.0282 (7)	
N5	0.6786 (2)	0.5834 (2)	0.2120 (3)	0.0376 (8)	
N6	0.5319 (2)	0.7455 (2)	0.3250 (3)	0.0398 (8)	
C1	1.0605 (2)	0.7434 (2)	0.0551 (3)	0.0250 (8)	
C2	1.1125 (2)	0.6297 (2)	0.0511 (3)	0.0319 (8)	
C3	1.2139 (2)	0.6250 (2)	0.0029 (4)	0.0344 (9)	
C4	1.2622 (2)	0.7337 (2)	−0.0353 (4)	0.0355 (9)	
C5	1.2058 (2)	0.8444 (2)	−0.0294 (3)	0.0307 (8)	
C6	0.9528 (2)	0.7623 (2)	0.1032 (3)	0.0257 (8)	
C7	0.8009 (2)	0.7528 (2)	0.1784 (3)	0.0262 (8)	
C8	0.6998 (2)	0.7053 (2)	0.2278 (3)	0.0258 (8)	
C9	0.6261 (2)	0.7845 (2)	0.2850 (4)	0.0328 (9)	
C10	0.5123 (3)	0.6236 (3)	0.3091 (4)	0.0420 (10)	
C11	0.5841 (3)	0.5442 (3)	0.2539 (4)	0.0446 (10)	
H2	1.07980	0.55750	0.08060	0.0380*	
H3	1.24950	0.54900	−0.00390	0.0410*	
H4	1.33200	0.73230	−0.06470	0.0430*	
H5	1.23870	0.91770	−0.05530	0.0370*	
H9	0.64390	0.86880	0.29570	0.0390*	
H10	0.44770	0.59120	0.33650	0.0500*	
H11	0.56640	0.45980	0.24520	0.0530*	

### Atomic displacement parameters (Å^2^)

**Table:**

	*U*^11^	*U*^22^	*U*^33^	*U*^12^	*U*^13^	*U*^23^
Cu1	0.0274 (3)	0.0210 (2)	0.0417 (3)	0.0025 (2)	0.0166 (2)	0.0069 (2)
N1	0.0255 (11)	0.0248 (11)	0.0306 (12)	−0.0007 (9)	0.0092 (9)	0.0017 (9)
N2	0.0296 (12)	0.0218 (11)	0.0384 (13)	0.0016 (9)	0.0150 (10)	0.0034 (9)
N3	0.0308 (12)	0.0241 (11)	0.0392 (13)	−0.0009 (10)	0.0174 (10)	0.0027 (10)
N4	0.0278 (12)	0.0226 (11)	0.0364 (13)	−0.0014 (9)	0.0127 (10)	0.0008 (9)
N5	0.0386 (14)	0.0235 (11)	0.0580 (16)	−0.0004 (11)	0.0257 (12)	−0.0021 (11)
N6	0.0340 (14)	0.0332 (13)	0.0579 (16)	0.0034 (11)	0.0222 (12)	−0.0018 (11)
C1	0.0237 (13)	0.0249 (13)	0.0252 (13)	−0.0014 (11)	0.0051 (11)	0.0005 (11)
C2	0.0345 (15)	0.0234 (13)	0.0378 (15)	−0.0019 (12)	0.0104 (12)	−0.0004 (11)
C3	0.0341 (16)	0.0282 (14)	0.0429 (16)	0.0073 (12)	0.0142 (13)	−0.0013 (12)
C4	0.0304 (15)	0.0365 (15)	0.0433 (17)	0.0050 (13)	0.0166 (14)	0.0024 (13)
C5	0.0262 (14)	0.0270 (14)	0.0408 (16)	−0.0008 (11)	0.0127 (12)	0.0046 (12)
C6	0.0269 (14)	0.0204 (13)	0.0301 (15)	−0.0008 (11)	0.0085 (12)	0.0019 (10)
C7	0.0287 (14)	0.0231 (12)	0.0282 (14)	−0.0015 (11)	0.0106 (12)	−0.0003 (10)
C8	0.0279 (14)	0.0226 (13)	0.0279 (14)	−0.0006 (11)	0.0097 (11)	0.0022 (11)
C9	0.0350 (16)	0.0233 (13)	0.0428 (16)	−0.0003 (12)	0.0156 (13)	−0.0003 (12)
C10	0.0337 (16)	0.0381 (16)	0.060 (2)	−0.0052 (13)	0.0225 (15)	0.0020 (14)
C11	0.0429 (18)	0.0256 (14)	0.073 (2)	−0.0053 (13)	0.0291 (17)	0.0016 (14)

### Geometric parameters (Å, º)

**Table:**

Cu1—N1	2.027 (2)	C1—C2	1.377 (3)
Cu1—N2	1.969 (2)	C1—C6	1.461 (3)
Cu1—N1^i^	2.027 (2)	C2—C3	1.376 (4)
Cu1—N2^i^	1.969 (2)	C3—C4	1.377 (3)
N1—C1	1.354 (3)	C4—C5	1.376 (3)
N1—C5	1.341 (3)	C7—C8	1.468 (3)
N2—N3	1.366 (3)	C8—C9	1.394 (3)
N2—C6	1.343 (3)	C10—C11	1.371 (5)
N3—C7	1.334 (3)	C2—H2	0.9300
N4—C6	1.332 (3)	C3—H3	0.9300
N4—C7	1.360 (3)	C4—H4	0.9300
N5—C8	1.334 (3)	C5—H5	0.9300
N5—C11	1.337 (4)	C9—H9	0.9300
N6—C9	1.325 (4)	C10—H10	0.9300
N6—C10	1.332 (4)	C11—H11	0.9300
			
N1—Cu1—N2	81.43 (9)	N4—C6—C1	129.0 (2)
N1—Cu1—N1^i^	180.00	N3—C7—N4	114.8 (2)
N1—Cu1—N2^i^	98.58 (9)	N3—C7—C8	121.6 (2)
N1^i^—Cu1—N2	98.58 (9)	N4—C7—C8	123.5 (2)
N2—Cu1—N2^i^	180.00	N5—C8—C7	117.7 (2)
N1^i^—Cu1—N2^i^	81.43 (9)	N5—C8—C9	120.7 (2)
Cu1—N1—C1	113.74 (17)	C7—C8—C9	121.6 (2)
Cu1—N1—C5	128.07 (16)	N6—C9—C8	123.2 (2)
C1—N1—C5	118.0 (2)	N6—C10—C11	122.3 (3)
Cu1—N2—N3	139.18 (16)	N5—C11—C10	122.5 (3)
Cu1—N2—C6	113.69 (18)	C1—C2—H2	121.00
N3—N2—C6	106.66 (19)	C3—C2—H2	121.00
N2—N3—C7	104.0 (2)	C2—C3—H3	120.00
C6—N4—C7	100.83 (19)	C4—C3—H3	120.00
C8—N5—C11	116.0 (2)	C3—C4—H4	120.00
C9—N6—C10	115.3 (3)	C5—C4—H4	120.00
N1—C1—C2	122.4 (2)	N1—C5—H5	119.00
N1—C1—C6	112.9 (2)	C4—C5—H5	119.00
C2—C1—C6	124.7 (2)	N6—C9—H9	118.00
C1—C2—C3	118.7 (2)	C8—C9—H9	118.00
C2—C3—C4	119.4 (2)	N6—C10—H10	119.00
C3—C4—C5	119.1 (2)	C11—C10—H10	119.00
N1—C5—C4	122.4 (2)	N5—C11—H11	119.00
N2—C6—N4	113.6 (2)	C10—C11—H11	119.00
N2—C6—C1	117.4 (2)		
			
N2—Cu1—N1—C1	8.16 (18)	C7—N4—C6—N2	0.2 (3)
N2^i^—Cu1—N1—C1	−171.84 (18)	C6—N4—C7—C8	177.0 (2)
N2—Cu1—N1—C5	−177.6 (2)	C8—N5—C11—C10	0.3 (4)
N2^i^—Cu1—N1—C5	2.4 (2)	C11—N5—C8—C7	−178.3 (2)
N1—Cu1—N2—N3	−178.5 (3)	C11—N5—C8—C9	0.0 (4)
N1^i^—Cu1—N2—N3	1.5 (3)	C9—N6—C10—C11	−0.5 (4)
N1—Cu1—N2—C6	−7.77 (18)	C10—N6—C9—C8	0.8 (4)
N1^i^—Cu1—N2—C6	172.23 (18)	N1—C1—C6—N2	0.5 (3)
Cu1—N1—C5—C4	−172.0 (2)	N1—C1—C2—C3	0.4 (4)
C1—N1—C5—C4	2.1 (4)	C2—C1—C6—N4	1.1 (4)
Cu1—N1—C1—C2	172.57 (18)	C2—C1—C6—N2	−179.0 (2)
C5—N1—C1—C2	−2.3 (4)	N1—C1—C6—N4	−179.5 (2)
Cu1—N1—C1—C6	−6.9 (3)	C6—C1—C2—C3	179.8 (2)
C5—N1—C1—C6	178.2 (2)	C1—C2—C3—C4	1.7 (4)
N3—N2—C6—C1	−179.9 (2)	C2—C3—C4—C5	−2.0 (4)
Cu1—N2—C6—N4	−173.66 (17)	C3—C4—C5—N1	0.0 (4)
Cu1—N2—N3—C7	170.9 (2)	N3—C7—C8—N5	162.8 (2)
C6—N2—N3—C7	−0.2 (3)	N4—C7—C8—C9	167.4 (2)
N3—N2—C6—N4	0.0 (3)	N4—C7—C8—N5	−14.3 (4)
Cu1—N2—C6—C1	6.4 (3)	N3—C7—C8—C9	−15.5 (4)
N2—N3—C7—C8	−177.0 (2)	C7—C8—C9—N6	177.6 (2)
N2—N3—C7—N4	0.3 (3)	N5—C8—C9—N6	−0.6 (4)
C7—N4—C6—C1	−179.9 (2)	N6—C10—C11—N5	−0.1 (5)
C6—N4—C7—N3	−0.3 (3)		

Symmetry code: (i) −*x*+2, −*y*+2, −*z*.

### Hydrogen-bond geometry (Å, º)

**Table:**

*D*—H···*A*	*D*—H	H···*A*	*D*···*A*	*D*—H···*A*
C3—H3···N5^ii^	0.93	2.52	3.303 (3)	141
C5—H5···N3^i^	0.93	2.39	3.169 (3)	141

Symmetry codes: (i) −*x*+2, −*y*+2, −*z*; (ii) −*x*+2, −*y*+1, −*z*.
